# Key Influences on Oral Feeding Achievement in Preterm Infants: Insights From a Tertiary Hospital in Indonesia

**DOI:** 10.1155/2024/8880297

**Published:** 2024-09-16

**Authors:** Putri Maharani Tristanita Marsubrin, Ni Nyoman Berlian Aryadevi, Bernie Endyarni Medise, Yoga Devaera

**Affiliations:** ^1^ Department of Child Health Faculty of Medicine Universitas Indonesia Dr. Cipto Mangunkusumo Hospital, Jakarta, Indonesia; ^2^ Department of Child Health Universitas Indonesia Hospital, Depok, Indonesia; ^3^ Faculty of Medicine Universitas Indonesia, Jakarta, Indonesia

**Keywords:** enteral nutrition, feeding methods, neonates, premature

## Abstract

**Objective**: Effective oral feeding is one of the critical milestones that must be achieved by preterm infants. While gestational age and birth weight have been recognized as influential factors, recent studies have found additional variables impacting the achievement of full oral feeding (FOF). This study is aimed at describing factors associated with the attainment of FOF in preterm infants.

**Methods**: This retrospective cohort study examines preterm infants born between 28 and 34 weeks' gestation admitted to Dr. Cipto Mangunkusumo General Hospital in Jakarta between July and December 2016. Comparative analysis utilized the Kruskal–Wallis test, while Cox's regression was employed for multivariate analysis to assess factors influencing the achievement of FOF.

**Results**: This study included 87 preterm infants meeting the inclusion criteria. The median gestational age was 33 weeks (IQR: 3). The most common birth weight range was 1500–1999 g (51.7%). Median durations from birth to the first feed, full enteral feed, and FOF were observed to be 1 day (IQR: 1), 6 days (IQR: 10), and 14 days (IQR: 24), respectively. Notably, the duration of oxygen therapy, episodes of sepsis, and frequency of blood transfusions showed significant associations with the time taken to achieve FOF.

**Conclusion**: This study found significant associations between the time to achieve FOF and factors such as oxygen therapy duration, sepsis episodes, and frequency of blood transfusion. These findings highlight the importance of considering these factors in managing preterm infants. However, a further prospective study is warranted to identify additional factors that influence feeding milestones in preterm infants.

## 1. Introduction

Full oral feeding (FOF) is a key indicator of preterm infants' readiness for discharge and overall maturity [1]. However, preterm infants are particularly vulnerable to feeding failure due to immature suck–swallow–breath coordination. Prematurity disrupts the development of suck central pattern generator (sCPG), a specialized neural circuit important for feeding skills as well [2].

Preterm infants are at heightened risk of respiratory problems, such as respiratory distress syndrome and bronchopulmonary dysplasia, due to their underdeveloped lungs. They are also more susceptible to other serious conditions, including intraventricular hemorrhage, necrotizing enterocolitis (NEC), and periventricular leukomalacia. These conditions result in prolonged oxygen supplementation thereby impending the initiation of oral feeding [3, 4]. The immaturity of their gastrointestinal tract and immune system predisposes them to infection. In cases of sepsis, preterm infants experience prolonged periods of fasting, reliance on parenteral nutrition, and delayed attainment of full enteral nutrition [5]. Consequently, the delay of oral feeding extends their hospitalization duration, leading to increased medical expenses [6].

In the discharge planning, it is important to understand the factors influencing the time taken to achieve FOF. Earlier research has identified factors like gestational age and birth weight as key influencers for achieving FOF. Though there is still some debate, recent studies have discovered other variables as well [6, 7]. This study is aimed at identifying factors associated with the attainment of FOF in preterm infants in Indonesia.

## 2. Methods

This is a retrospective cohort study on preterm infants hospitalized in Cipto Mangunkusumo (CM) Hospital, Jakarta, between July and December 2016. Utilizing secondary data obtained from a previously published study titled “Growth and Development of 28–34 Weeks of Gestational Age Premature Infants Post-Discharge,” [8] data collection from medical records took place between July 2016 and May 2017. The ethical approval for this study was obtained from the CM Hospital ethical committee (No. 267/UN2.F1/ETIK/2016).

### 2.1. Participants

Preterm infants within the gestational age range of 28–34 weeks, residing in Jakarta, Bogor, Depok, Tangerang, and Bekasi, were included with parental consent. Exclusions comprised infants with congenital abnormalities, syndromes, and orofacial malformations and those lacking oral feeding records. Consecutive sampling was employed for participant recruitment.

Twelve predictors were collected, including gestational age, birth weight, APGAR scores at the first and fifth minutes postbirth, hyperbilirubinemia, anemia, NEC, sepsis, number of sepsis episodes, oxygen therapy use and duration, and frequency of blood transfusions. Data collection criteria were as follows:

• Gestational age: recorded in weeks
• Birth weight: categorized as < 1500, 1500–1999, and ≥ 2000 g
• NEC diagnosis: as per Bell's criteria
• Sepsis diagnosis: adhered to NICE guidelines on neonatal infection [9], with sepsis episodes categorized as zero, one time, and ≥ 2 times
• Oxygen therapy duration: recorded in days, with < 24 h rounded as 1 day
• Blood transfusions: based on Hb threshold/goals [10] and clinical judgment
• Hyperbilirubinemia: diagnosed according to local guidelines

The attainment of the first feed is characterized by the duration from birth to the initiation of the initial feed, regardless of the route or volume administered. On the other hand, the time to reach full enteral feed is defined as the duration from birth to the feeding of 150 mL/kg, sustained for 24 h. The primary outcome measure was the time to achieve FOF, defined as the duration from birth until all feeds were consumed orally or the removal of the nasogastric tube. APGAR scores were calculated in the first (APGAR-1) and fifth (APGAR-5) minutes after birth.

### 2.2. Statistical Analysis

Descriptive statistics, normality tests, comparative analyses, and multivariate analyses were conducted using IBM SPSS Version 24.0 (SPSS Inc, Chicago, Illinois). Due to the nonnormal distribution of predictor variables, the Kruskal–Wallis test was selected for comparative analysis, while Cox's regression was employed for multivariate analysis to ascertain the contributions of predictors to the primary outcome.

## 3. Results

**Figure 1:**
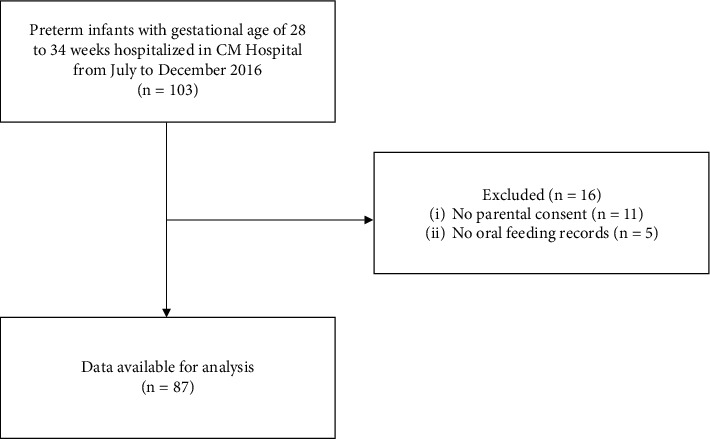
Flow diagram of participant recruitment.

Among the 103 preterm infants admitted to CM Hospital between July and December 2016, with gestational ages ranging from 28 to 34 weeks, 11 were excluded due to parental nonconsent, and an additional five were excluded because of the absence of oral feeding records (Figure 1).

In this study, 42 female and 45 male infants were included. The majority of the infants were born with gestational ages of 33 weeks (25.3%) and 34 weeks (25.3%), followed closely by 31 weeks (20.7%) and 32 weeks (18.4%). The median gestational age was 33 weeks (IQR: 3). A predominant proportion of preterm infants fell within the birth weight range of 1500–1999 g (51.7%). Hyperbilirubinemia was observed in 57 participants (65.5%), while 20 participants (23%) experienced anemia and nine participants (10.3%) were diagnosed with NEC. Sepsis was prevalent among 67.8% of participants, with the majority experiencing a single episode (59.8%), while seven participants had more than two episodes (Table 1). Oxygen therapy was administered to 66 participants, with a median duration of 1 day (IQR: 8). Median APGAR-1 and APGAR-5 scores were 7 (IQR: 2) and 9 (IQR: 1), respectively.

**Figure 2:**
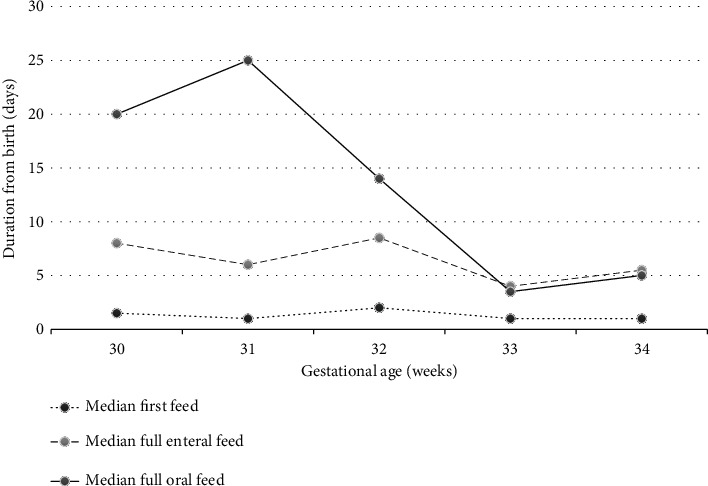
Median duration to the first feed, full feed, and FOF from birth in days.

The median durations from birth to the first feed, full enteral feed, and FOF for all infants were 1 day (IQR: 1), 6 days (IQR: 10), and 14 days (IQR: 24), respectively (Table 2). The median duration to attain FOF increased from 20 days in the 30-week gestational age group to 25 days in the 31-week gestational age group. Subsequently, it decreased from the 31-week gestational age group to the 33-week gestational age group, followed by a modest increase of 1.5 days in the 34-week gestational age group (Figure 2).

Statistically significant differences in the duration to achieve FOF were observed across various predictors, including gestational age, the presence of sepsis, anemia, and the administration of oxygen therapy. Furthermore, differences in the time to attain FOF were significant concerning APGAR-1 and APGAR-5 scores, frequency of transfusions, sepsis episodes, and birth weight groups (Table S1).

A 1-day increase in oxygen therapy is significantly associated with a 9% higher likelihood of delayed attainment of FOF compared to individuals who did not undergo oxygen therapy. Moreover, participants with two or more sepsis episodes exhibit a 97% increased probability (*p* value: 0.011) of reaching FOF later, in contrast to those with no sepsis occurrences. Additionally, those who had more than two frequencies of blood transfusion had 99% increased probability (*p* value: 0.003) of attaining FOF later compared to those who never had any blood transfusion (Table 3).

## 4. Discussion

The primary goal of preterm infant nutrition is to achieve growth and developmental rates comparable to those in utero [11]. To achieve adequate growth, the establishment of effective oral feeding is needed. Furthermore, achieving independent oral feeding is a key criterion for hospital discharge in infants [12]. Despite its importance, achieving this milestone remains one of the most common challenges to discharging preterm infants.

In our study, the average time to start enteral feedings was 1 day, which aligns with ESPGHAN guidelines that recommend starting enteral nutrition on the first day of life [13]. A meta-analysis revealed that early enteral feeding (< 72 h) is associated with decreased mortality risk, shorter hospital stays, and potentially lower sepsis incidence [14]. Despite ongoing concerns about early enteral feeding and its potential association with NEC, evidence suggests that it has minimal effect on NEC incidence [14]. The median time to attain full enteral feeding was 6 days in our study. Rapid attainment of full enteral nutrition within 5–7 days postbirth has been associated with achieving growth rates similar to intrauterine trajectories, even among extremely preterm infants [15, 16].

The median duration to achieve FOF in our study was 14 days, consistent with findings from a New Zealand study with a median duration of 12 days [17]. While previous research on preterm infants with comparable gestational ages has shown transition times from initial oral feeding to FOF ranging from 13 to 16 days, time to reach FOF is not easy to compare between studies due to differences in definitions; some measure it from the first oral feed, while others start from the day of birth [18, 19]. Our analysis shows that infants born at earlier gestational ages generally take longer to reach FOF. Interestingly, the median time to achieve FOF was longer for infants born at 31 weeks compared to those born at 30 weeks. This difference may be related to the higher prevalence of very-low-birth-weight infants in the 31-week group. Specifically, out of 32 infants with a birth weight under 1500 g, 15 were born at 31 weeks.

Gestational age has been demonstrated to influence maturation of feeding skills [20]. Earlier studies suggested that preterm infants could safely initiate oral feeding at 34 weeks' gestational age, based on their coordinated sucking, swallowing, and breathing abilities, while recent research emphasizes on additional factors, such as nonnutritive sucking ability, in the decision to start oral feeding. Consequently, safe oral feeding may be considered even in infants younger than 34 weeks' gestational age [19]. There are factors associated with independent oral feeding, including birth weight, sex, and health status. A negative correlation has been observed between the development of oral feeding, lower birth weight, and increased medical complications [6, 21, 22]. Feeding difficulties are common in infants with low birth weight, due to immature oromotor skills and a lack of coordination between sucking, swallowing, and breathing [23].

Our findings indicate significant associations between oxygen therapy duration, episodes of sepsis, and frequency of blood transfusion with the time required to achieve FOF. This aligns with the results reported by Jiménez et al., who reported correlations between the age at FOF and the duration of oxygen therapy, sepsis incidence, and transfusion events [7]. However, our study revealed that only individuals experiencing more than two episodes of sepsis and two episodes of blood transfusion exhibited a significantly prolonged time to achieve FOF. Other than their immature cardiorespiratory function contributing to oral feeding problems, prolonged oxygen therapy may also disrupt the coordination of breathing, sucking, and swallowing rhythms essential for coordinated suckling [7, 24]. Similar to our study, De Sousa et al. reported that neonates with sepsis took longer to achieve full enteral nutrition compared to those without sepsis. This prolonged duration also heightened the risk of developing late-onset sepsis [5]. In neonates requiring > 10 days to attain full enteral feeding, the incidence of late-onset sepsis is notably higher [25].

Being a retrospective study utilizing secondary data, our research is subject to certain limitations. Some variables pertinent to the achievement of oral feeding were not accessible for collection, including the number of feeds administered before reaching FOF, nonnutritive sucking, and maternal factors. Consequently, the comprehensive understanding of factors influencing oral feeding attainment may be incomplete. To comprehensively identify all key predictors, further prospective studies are warranted.

## Figures and Tables

**Table 1:** Characteristics of participants.

	**n**	**%**
Gestational age (weeks)		
29	1	1.1%
30	8	9.2%
31	18	20.7%
32	16	18.4%
33	22	25.3%
34	22	25.3%
Sex		
Male	45	51.7%
Female	42	48.3%
Birth weight (g)		
< 1500	30	34.5%
1500–1999	45	51.7%
≥ 2000	12	13.8%
Hyperbilirubinemia	57	65.5%
Anemia	20	23%
Blood transfusion frequency		
0	67	32.2%
1×	13	59.8%
≥2×	7	8%
NEC	9	10.3%
Sepsis	59	67.8%
Sepsis episodes		
0	28	32.2%
1×	52	59.8%
≥ 2×	7	8%
Oxygen therapy	66	75.9%

Abbreviation: NEC: necrotizing enterocolitis.

**Table 2:** Median duration to the first feed, full enteral feed, and FOF from birth in days.

**Gestational age (weeks)**	**N**	**Median first feed (days)**	**IQR**	**Median full enteral feed (days)**	**IQR**	**Median FOF (days from birth)**	**IQR**
29	1	2		21			
30	8	1.5	2.5	8	4.75	20	25
31	18	1	1	6	12.75	25	20
32	16	2	6.75	8.5	13.75	14	25
33	22	1	0	4	8.25	3.5	15
34	22	1	2.25	5.5	8.5	5	16

Abbreviations: FOF: full oral feed; IQR: interquartile range.

**Table 3:** Multivariate results of predictors in duration to attain FOF from birth.

**Predictors**	**HR [95% CI]**	**p** ** value**
Oxygen therapy duration	0.913 [0.873, 0.954]	
Sepsis episodes		
0		0.007
1	0.284 [0.32, 2.523]	0.259
≥ 2	0.031 [0.002, 0.446]	0.011
Frequency of blood transfusion		
0		0.010
1	0.222 [0.047, 1.051]	0.058
≥ 2	0.003 [0.010, 0.379]	0.003

Abbreviations: CI: confidence interval; HR: hazard ratio.

## Data Availability

The data that support the findings of this study are available on request from the corresponding author. The data are not publicly available due to privacy or ethical restrictions.
