# Supplementary material for: Key Influences on Oral Feeding Achievement in Preterm Infants: Insights From a Tertiary Hospital in Indonesia
Source: Int J Pediatr. 2024 Sep 16;2024:8880297. doi: 10.1155/2024/8880297 (PMC11419833; doi:10.1155/2024/8880297)
Supplement: Supporting Information — Additional supporting information can be found online in the Supporting Information section. Table S1. Kruskal–Wallis comparison of duration to reach FOF from birth. [file 8880297.f1.docx]

**Supplementary data**

**Supplementary Table 1.** Kruskal-Wallis comparison of duration to reach FOF from birth

| Predictors | H | | d.f |
| --- | --- | --- | --- |
| Gestational age | | 14.92* | 5 |
| Sepsis | | 17.85* | 1 |
| Oxygen therapy | | 11.94* | 1 |
| Hyperbilirubinemia | | 0.022 | 1 |
| APGAR-1 | | 18.58* | 7 |
| APGAR-5 | | 12.67* | 5 |
| Sepsis episode | | 27.68* | 2 |
| Anemia | | 27.98* | 1 |
| Blood transfusion frequency | | 27.89* | 2 |
| NEC | | 2.93 | 1 |
| Birth weight | | 16.07* | 2 |

*p-value < 0.05
